# Self-Assembly Stability and Variability of Bacterial Microcompartment Shell Proteins in Response to the Environmental Change

**DOI:** 10.1186/s11671-019-2884-3

**Published:** 2019-02-12

**Authors:** Matthew Faulkner, Long-Sheng Zhao, Steve Barrett, Lu-Ning Liu

**Affiliations:** 1 Institute of Integrative Biology, University of Liverpool, L69 7ZB, Liverpool, UK; 2 Department of Physics, University of Liverpool, L69 7ZE, Liverpool, UK

**Keywords:** Bacterial microcompartment, Protein dynamics, Self-assembly, High-speed atomic force microscopy, Synthetic engineering

## Abstract

Bacterial microcompartments (BMCs) are proteinaceous self-assembling organelles that are widespread among the prokaryotic kingdom. By segmenting key metabolic enzymes and pathways using a polyhedral shell, BMCs play essential roles in carbon assimilation, pathogenesis, and microbial ecology. The BMC shell is composed of multiple protein homologs that self-assemble to form the defined architecture. There is tremendous interest in engineering BMCs to develop new nanobioreactors and molecular scaffolds. Here, we report the quantitative characterization of the formation and self-assembly dynamics of BMC shell proteins under varying pH and salt conditions using high-speed atomic force microscopy (HS-AFM). We show that 400-mM salt concentration is prone to result in larger single-layered shell patches formed by shell hexamers, and a higher dynamic rate of hexamer self-assembly was observed at neutral pH. We also visualize the variability of shell proteins from hexameric assemblies to fiber-like arrays. This study advances our knowledge about the stability and variability of BMC protein self-assemblies in response to microenvironmental changes, which will inform rational design and construction of synthetic BMC structures with the capacity of remodeling their self-assembly and structural robustness. It also offers a powerful toolbox for quantitatively assessing the self-assembly and formation of BMC-based nanostructures in biotechnology applications.

**Electronic supplementary material:**

The online version of this article (10.1186/s11671-019-2884-3) contains supplementary material, which is available to authorized users.

## Introduction

**Fig. 1:**
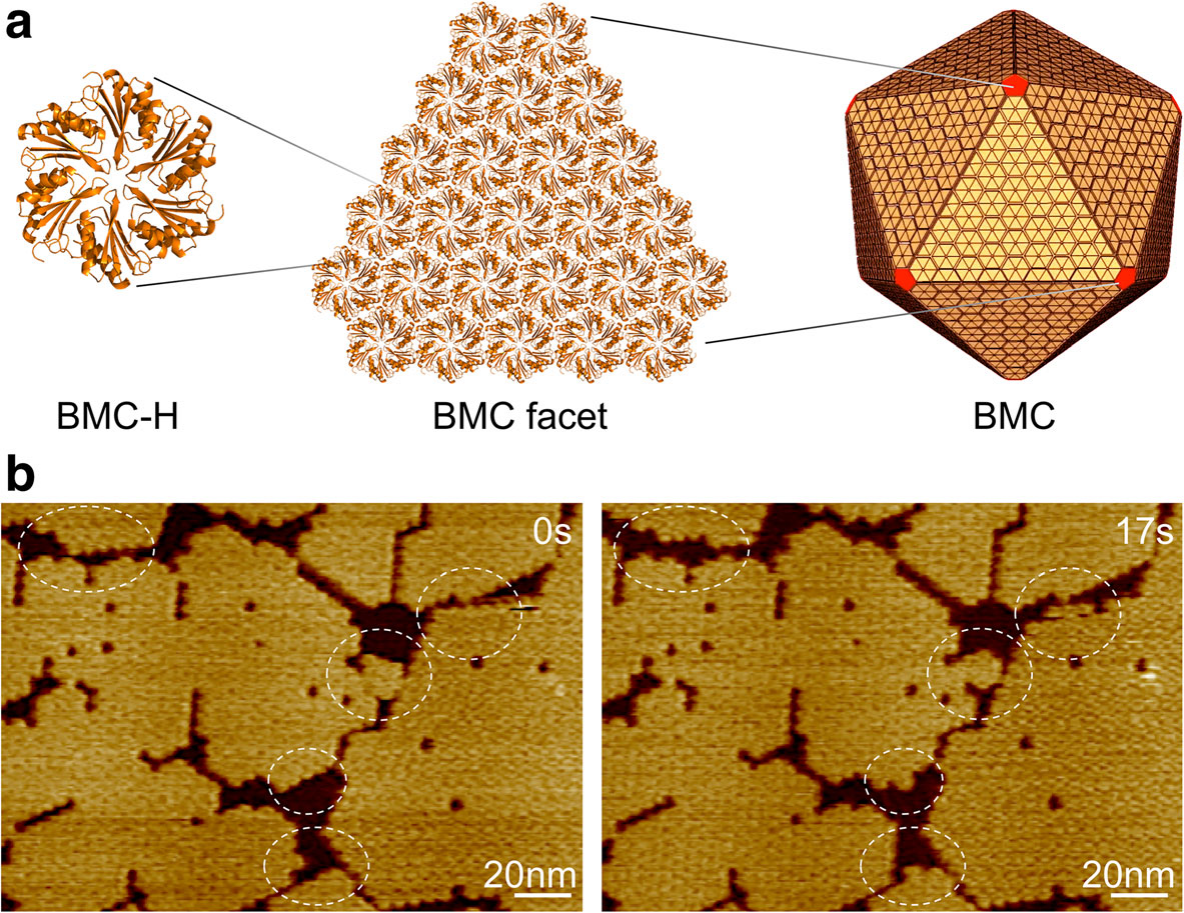
Bacterial microcompartment, shell organization, and self-assembly. **a** Hundreds of copies of BMC shell protein homologs self-assemble to form an icosahedral protein organelle. BMC-H proteins, in yellow, form the facets and BMC-P proteins, in red, occupy the vertices. **b** AFM topographs of shell facets composed of Hoch_5815 BMC-H hexamers. Dynamic events (circles) were observed within seconds using HS-AFM

Bacterial microcompartments (BMCs) are proteinaceous organelles, structurally resembling viral capsids, that partition the cytoplasm of bacteria [1]. They are widespread among bacterial phyla [2] and allow bacteria to compartmentalize key metabolic pathways in the absence of membrane-bound organelles found in eukaryotes [3, 4]. BMCs are formed by a semi-permeable protein shell encapsulating a luminal enzyme core. The shell is composed of three types of structural protein components, including BMC-H (containing a Pfam00936 domain), BMC-T (containing two Pfam00936 domains), and BMC-P (with a Pfam03319 domain) [5–9]. The major components of the shell are BMC-H, which appear as hexamers with convex and concave surfaces and tile the shell facets with their concave side facing outward [10] (Fig. 1). BMC-P form pentamers that are proposed to cap the vertices of the icosahedral shape, and BMC-T form pseudohexamers that are located in the shell facets presumably responsible for shell permeability.

Specific protein-protein interactions ensure the self-assembly of BMC proteins to form highly defined architectures to fulfill their metabolic functionality. The lateral interactions between shell proteins are assumed to be the major factor for determining the self-assembly properties of the icosahedral shell [10]. It has been observed that BMC-H homologs can form various shapes, including two-dimensional sheets [11, 12], nanotubes [13–17], and filament structures [15, 18–20].

Based on the self-assembly, selective permeability and enzyme encapsulation properties of the naturally occurring organelles, BMCs have been considered as an ideal system with great potential in bioengineering, including bioinspired construction of nanoscale bioreactors by encasing metabolic enzymes and generation of new molecular scaffolds with new functions [21–26]. However, some key issues remain to be tackled in BMC bioengineering, for example, how stable the BMC structures are and how to manipulate and assess effectively the self-assembly and formation of BMC protein aggregates. Investigations of the structures and assembly of BMC shells and entire BMCs have been carried out using X-ray crystallography, electron microscopy (EM), fluorescence microscopy, and dynamic light scattering (DSL) [10, 11, 16, 22, 27–31]. Recently, we have exploited high-speed AFM (HS-AFM) to conduct the first visualization of the dynamic self-assembly process of BMC-H proteins [12].

In this work, we use HS-AFM to monitor the structural dynamics of BMC-H patches under varying pH and ionic conditions, which provides insight into the modulation of BMC shell protein assembly and offers a powerful tool for quantitative assessment, at the molecular resolution, on the stability and variability of BMC shell protein self-assembly.

## Methods

### Sample Preparation

The purified BMC-H protein (Hoch_5815) from *Haliangium ocraceum* was kindly provided by Dr. Kerfeld (Lawrence Berkeley National Laboratory). For buffer exchange, stock samples at ~ 80 mg mL^−1^ in Tris buffer (50 mM Tris-HCl, pH 7.8, 100 mM KCl, 10 mM MgCl_2_) were diluted to 0.5 mg mL^−1^ using the desired buffer prior to AFM imaging (Additional file 1: Figure S1). The control buffer is 50-mM Tris-HCl (pH 7.8) and 10 mM MgCl_2_.

### Atomic Force Microscopy

Desired buffers were used for sample absorption on mica and AFM imaging. After 5 min absorption on the mica, Hoch_5815 were rinsed with the desired buffer to remove immobilized proteins and then imaged using AFM (Additional file 1: Figure S1). HS-AFM images were captured at 30 or 40 Hz in solution in AC mode using a JPK NanoWizard ULTRA speed AFM equipped with an ULTRA Speed 2.8 μm scanner and Ultra-Short Cantilever USC-0.3 MHz probes (NanoWorld). Minimal loading forces of ~ 100 picoNewton were applied during AFM imaging to reduce disturbance of protein assembly [12, 32–36].

### Image Processing and Analysis

Image analysis was initially performed using JPK SPM Data Processing (JPK). HS-AFM image analysis was performed using a custom macro on Image SXM (http://www.ImageSXM.org.uk), as described previously [12]. To analyze the sizes of Hoch_5815 patches, images of 512 × 512 pixels captured at 30 Hz scan rate were flattened to remove any XY tilt and Z thresholded, followed by binary conversion to display protein versus not protein. Particle analysis was used to calculate the surface area of proteins in these binary images. Patches were defined as objects separated by > 3 pixels (~ 2 nm), in order to identify individual patches versus adjacent patches. Initial tests showed that if a larger number of pixels is set, adjacent patches could be counted as a single continuous patch, whereas using a smaller pixel number, the gaps between individual hexamers in patches could be miscounted as the boundary between patches. To analyze protein dynamics, image series of 256 × 256 pixels captured at 40-Hz scanning speed were analyzed giving a temporal resolution of approximately 6.4 s per frame. Binary images were subtracted from the previous image in the series to show difference AFM images. Particle analysis of the difference images was employed to count the area of assembled and disassembled proteins. The equation used for calculating the dynamic rate is shown as follows:

$$ \mathrm{Rate}\ \mathrm{of}\ \mathrm{dynamic}\ \mathrm{events}\ (R)=\frac{\mathrm{Number}\ \mathrm{of}\ \mathrm{hexamers}\ \mathrm{added}\ \mathrm{or}\ \mathrm{removed}\ \mathrm{in}\ \mathrm{a}\ \mathrm{s}\mathrm{eries}\ \mathrm{of}\ \mathrm{frame}\mathrm{s}\kern0.5em (N)}{\mathrm{Total}\ \mathrm{surface}\ \mathrm{area}\ \mathrm{of}\ \mathrm{protein}\ \mathrm{in}\ \mathrm{frame}\kern0.5em (A)\times \mathrm{time}\kern0.5em (T)}, $$

where *N* represents the sum of white and black pixels in a thresholded difference image divided by the number of pixels corresponding to a single hexamer at that scale (Additional file 1: Figure S3, Figure S5). Data is presented as mean ± standard deviation (SD). Statistical analysis was performed using multivariate ANOVA or two-way ANOVA as specified.

## Results

We used the BMC-H proteins (Hoch_5815) from a myxobacterium *Haliangium ocraceum*, which were expressed in *Escherichia coli* and characterized as hexamers with a six-fold symmetry [12]. Hoch_5815 hexamers could self-assemble to form single-layered sheets at the second timescale, which represent the basic structural components of the icosahedral BMC architecture (Fig. 1a). HS-AFM imaging allows us to visualize the dynamic assembly and organizational flexibility of sheet fragments (Fig. 1b) and quantitatively estimate the patch size and dynamic rate of BMC-H proteins using the developed imaging analysis (see the “Methods” section).

### Response to pH Variation

**Fig. 2:**
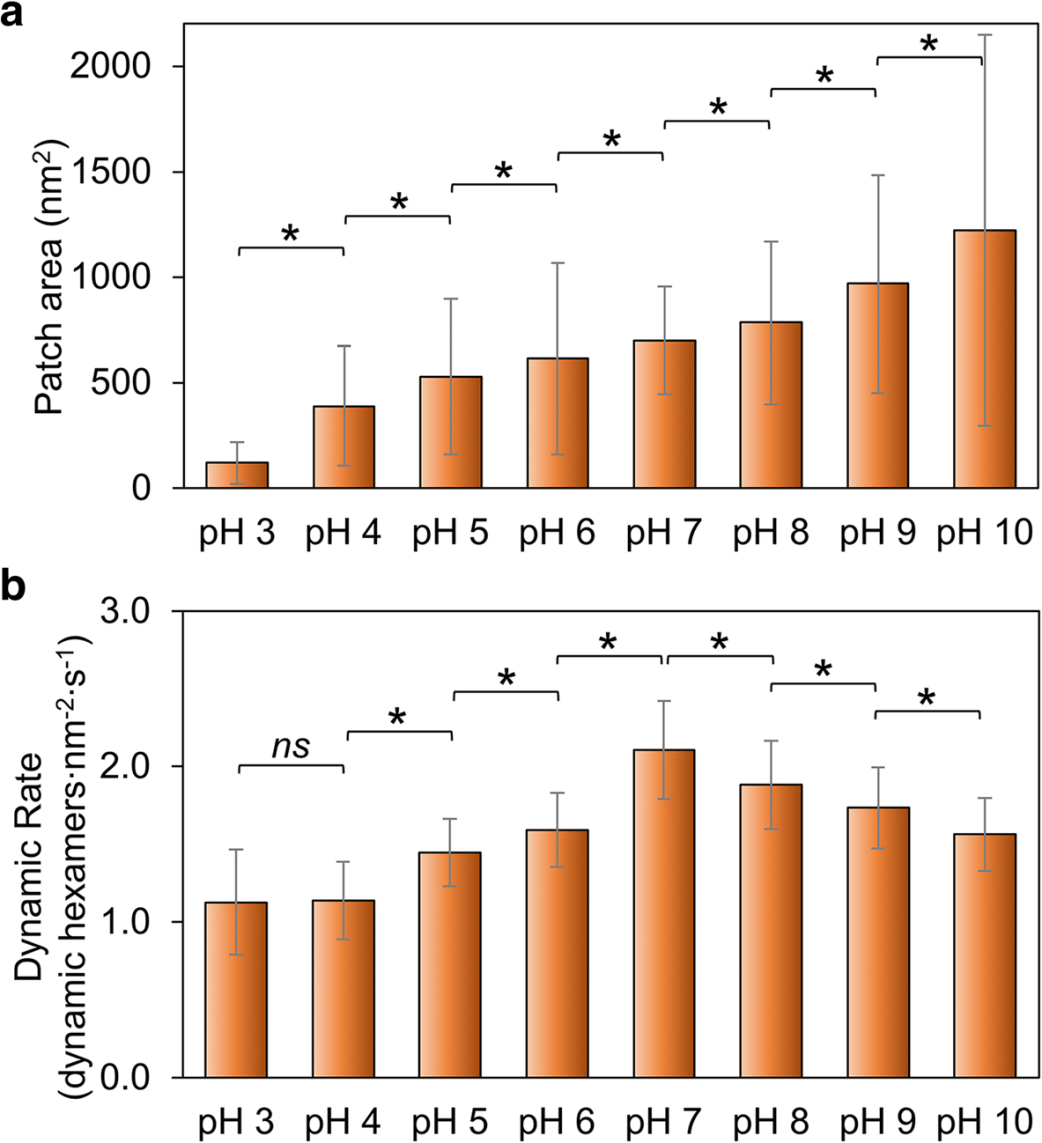
Effects of environmental pH on the self-assembly of Hoch_5815. **a** The mean surface areas of individual patches of Hoch_5815 determined by AFM (*n* = 50) (Additional file 1: Figure S2). **b** The mean rates of dynamic events determined by HS-AFM (*n* = 50). *p < 0.05, *ns* not significant (multivariate ANOVA)

We measured the changes in patch size as an indication of the overall ability of Hoch_5815 to self-assemble. The patch size increases with the rise of pH from 3 to 10 (Fig. 2a; Additional file 1: Figure S2, Table S1), suggesting that high pH is more favorable for the self-assembly of Hoch_5815 proteins than low pH conditions. This is somewhat distinct from the assembly behaviors of RmmH proteins, which were found to be insoluble at pH 6, form ordered arrays of nanotubes at pH 8, and were prone to disassembly at pH 10 [13]. In addition, we observed a high degree of structural variability of the HOCH_5815 self-assembles (as indicated by a large SD in Fig. 2a, Additional file 1: Figure S2).

AFM imaging on the self-assembly of Hoch_5815 proteins in shell sheets has revealed that the formation of shell sheets is ascribed to a combination of the assembly and disassembly of hexamers [12]. We further examined the rates of Hoch_5815 self-assembly dynamics and dynamic events under different pH (Additional file 1: Table S2) to explore the stability of Hoch_5815 protein-protein interactions. The rate of self-assembly dynamics is the highest at pH 7 and decreases in both acidic and alkaline conditions (Fig. 2b; Additional file 1: Figure S3). In particular, it declines rapidly in acidic conditions, notably from pH 7 to pH 6 and appears relatively constant between pH 4 and pH 3, as shown in Fig. 2b.

It is likely that pH has a great impact on the electrostatic properties of amino acid residues located at the hexamer-hexamer interface. The decreased dynamics and a smaller size of shell patches observed in acidic conditions illustrate that Hoch_5815 has a reduced self-assembling ability. The reduced dynamics and a larger size of shell patches observed in the alkaline conditions suggest stable hexamer-hexamer interactions, whereas the increased dynamics of Hoch_5815 hexamers imply flexible hexamer-hexamer interactions in the neutral pH condition.

### Response to the Variation of Salt Concentrations

**Fig. 3:**
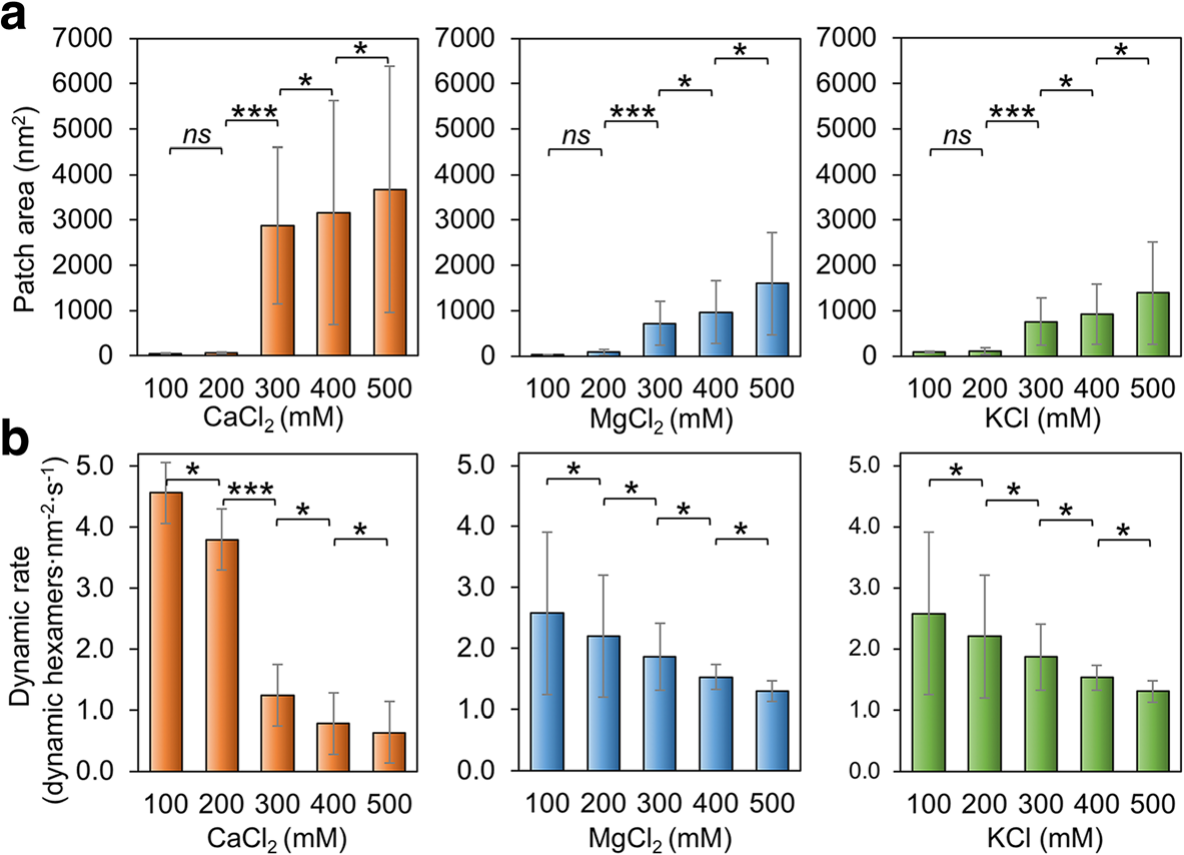
Effects of salt concentration on the self-assembly of Hoch_5815. **a** The mean patch areas measured by AFM under a range of 100–500 mM CaCl_2_, MgCl_2_, and KCl (*n* = 50). The rise in salt concentration resulted in increased patch sizes. Significant changes in patch area were observed between 200 and 300 mM (***p < 0.001, *p < 0.05, *ns* not significant, two-way ANOVA). **b** The mean rates of dynamic events determined from high-speed AFM image series under a range of 100–500 mM CaCl_2_, MgCl_2_, and KCl (*n* = 50). Each 100 mM change in salt concentration led to a significant change in the rate of dynamic events (***p < 0.001, *p < 0.05, *ns* not significant, two-way ANOVA)

We also verified if salt concentration of the buffer has impacts on the assembly of Hoch_5815. At low concentrations (100–200 mM) of MgCl_2_, CaCl_2_, and KCl, Hoch_5815 proteins form relatively smaller patches than those assembled at higher concentrations (300–500 mM) (Fig. 3a; Additional file 1: Figure S4). At 500 mM, we observed double- or multi-layered Hoch_5815 sheets (Additional file 1: Figure S4). These observations are consistent with the previous finding that higher ionic strength could facilitate the formation of more extensive and well-ordered 2D crystals by CcmK, the shell proteins of carboxysomes for carbon assimilation [37]. However, the highly ordered nanotubes formed by RmmH were disassembled when NaCl concentration was increased from 50 to 500 mM [13], indicating the potentially different mechanisms that mediate the formation of flat sheets and tubular shapes by shell hexamers.

Moreover, the variations of Hoch_5815 self-assembly caused by the changes in MgCl_2_ and KCl concentrations are relatively similar. By contrast, the change in patch size is most pronounced (up to a 3000-fold increase) when the CaCl_2_ concentration is raised from 200 to 300 mM (Fig. 3a), suggesting the higher sensitivity of Hoch_5815 self-assembly to CaCl_2_ than to MgCl_2_ or KCl.

The dynamic rate of Hoch_5815 self-assembly is also affected by changes in the buffer salt concentration. The increase in MgCl_2_, CaCl_2_, or KCl concentrations could result in the decline of the Hoch_5815 dynamic rate (Fig. 3b; Additional file 1: Figure S5). Given the increase in the patch size observed under higher salt concentrations (Fig. 3a), it appears that the lateral interactions between Hoch_5815 hexamers are more stable under high salt concentrations. Changes in CaCl_2_ concentration had a more pronounced response, and there was a significant shift in the rate of dynamic events between 200 and 300 mM (Fig. 3b), whereas the responses to the changes in MgCl_2_ and KCl are relatively similar, consistent with the changes in the patch size (Fig. 3a). Interestingly, the highest proportions of assembly events versus disassembly events were observed under 400 mM of MgCl_2_, CaCl_2_, or KCl (Additional file 1: Table S2). This led to the formation of large and stable single-layer Hoch_5815 assemblies under 400 mM salt (Additional file 1: Figure S4). The double-layer assemblies observed at 500 mM are also stable and exhibit low rates of hexamer movement.

### Flexibility of BMC-H Protein Assembly

**Fig. 4:**
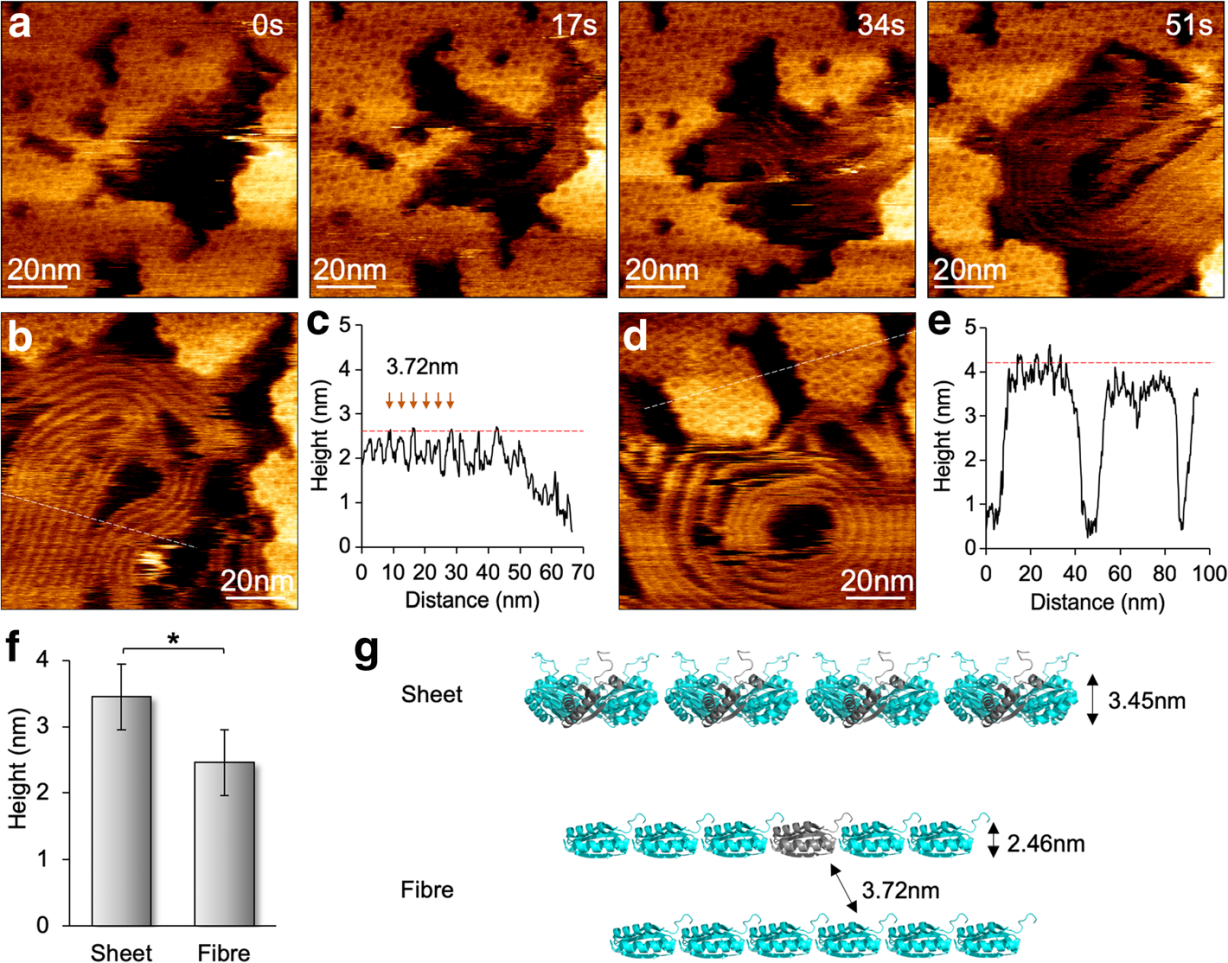
Formation and dynamics of fibrous structures along with the shell sheet assemblies under HS-AFM. **a** Appearance of fiber-like structures during the disassembly of shell sheets composed of Hoch_5815 hexamers, as shown by AFM image series. **b** AFM topograph of fiber structures. **c** Cross-section analysis (dash line in panel **b**) reveals a spacing of 3.72 ± 0.31 nm (*n* = 30) between adjacent fiber structures, and the average height is 2.46 ± 0.22 nm (*n* = 30). **d** AFM topograph of shell patches composed of Hoch_5815 hexamers. **e** Cross-section analysis (dash line in panel **d**) reveals that the average height of Hoch_5815 hexamers is 3.45 ± 0.16 nm (*n* = 25). **f** The fiber-like structures present a reduced height compared with flat sheets consisting of Hoch_5815 hexamers (*p < 0.05, two-way ANOVA). **g** Proposed organization and formation of the fiber-like structure, representing a string of Hoch_5815 monomers

By reducing the scanning force to 100 pN, we minimized the effects of AFM tip scanning on the assembly of BMC proteins and obtained molecular resolution AFM images of individual hexamers (Fig. 4). Both assembly and disassembly events can been seen in the same view, verifying the dynamic nature of BMC shell assembles instead of tip scanning artifacts [12]. HS-AFM imaging also revealed the variability of Hoch_5815 protein aggregations. When imaging the samples at pH 7.5 in the presence of only 10 mM MgCl_2_, surprisingly, we occasionally observed the formation of fiber-like structures along with the disassembly of Hoch_5815 hexamers at the second timescale (Fig. 4a). These fiber-like structures could be densely packed in parallel, similar to the nanotube bundles assembled by shell hexamers [13–16]. However, the space between two fibers is 3.72 ± 0.31 nm (*n* = 30) and their average height is 2.46 ± 0.22 nm (*n* = 30), less than that of shell sheets formed by Hoch_5815 hexamers (3.45 ± 0.16 nm, *n* = 25) (Fig. 4b–f). These fiber structures are fairly flexible and dynamic during imaging and could display straight or coiled architectures with different sizes. Given the concomitant appearance of the fiber structures with the disassembly of Hoch_5815 hexamers and their reduced height compared with the single-layer hexamer sheets, we speculate that these fiber-like structures are formed by the individual Hoch_5815 peptides disassembled from hexamers (Fig. 4g). It is likely that the substrate absorption in specific buffer conditions (such as low ionic strength) could lead to attachment of the alpha-helix sides of Hoch_5815 peptides to the substrate surface and the linear binding of the peptides with neighboring peptides, although it is assumed that intra-hexamer interactions are likely strong [5]. The detailed mechanism underlying the variability of shell protein aggregation remains to be further elucidated.

## Discussion

BMCs comprise hundreds of proteins that self-assemble to form the higher ordered structures. The BMC shell, consisting of numerous protein homologs, is an ideal system for studying protein self-assembly and interactions. As a powerful technique for analyzing biomembrane organization, protein assembly, and physical interactions that are highly relevant to the physiological roles of biological systems [32, 35, 38, 39], AFM has been exploited to visualize the organization and self-assembly dynamics of BMC shell proteins and the architectures and mechanical features of BMC structures [12, 30, 31, 40–42]. This work represents, to our knowledge, the first quantitative determination of the self-assembly dynamics of BMC shell proteins in the formation of two-dimensional sheets in response to environmental changes using AFM. The results highlight the inherent variability and environmental dependence of BMC-H protein self-assembly. Compared with EM and DSL, AFM exhibits great potential in monitoring the dynamic actions of BMC protein self-assembly in real time with molecular details.

Protein-protein interactions are of significant importance in forming and shaping the BMC shell [10]. The protein concentration has also been documented as a critical factor for driving shell formation [41, 43]. In addition, in vitro solubility studies have illustrated that pH and ionic strength in solution could influence the structural stability of BMCs [17, 27] as well as the assembly behaviors of BMC shell proteins in the formation of two-dimensional sheets [37, 41], nanotubes [13, 17], and nanocages [28], reminiscent of their impact on virus capsid assembly [44, 45]. We also found protein precipitation and no patches formed when pH > 10 and < 3 or the salt concentration < 10 mM or > 600 mM (unpublished data). Here, we further showed that the assembly tendency and dynamics are dependent on pH and salt concentration. Though shell proteins can self-assemble at a wide range of pH, the neutral pH environment appears to be capable of enhancing the assembly dynamics (Fig. 2b). Cations with a concentration of ≥ 300 mM were found to promote the formation of two-dimensional sheets; 400 mM cations appear to be desirable for the formation of large and stable single-layered sheets (Fig. 3). These conditions align with the cytosolic conditions of bacterial cells and are physiologically relevant. For example, under most physiologically relevant conditions, the pH of the *E. coli* cytosol is approximately 7.4–7.8 [46] and the ion concentration is approximately 100–400 mM, which is vital for protein interactions, protein-ligand binding, signaling, maintaining membrane electrostatic potentials, and protein gradient across membranes [47, 48]. Although how interactions between samples and the mica substrate affect the self-assembly of BMC proteins remains to be further investigated, AFM imaging provides the opportunity for us to quantitatively analyze the dynamic changes of BMC protein self-assembly in response to environmental variations.

The environment-dependent assembly dynamics of BMC proteins in the formation of shell fragments described here might represent their behaviors in the formation of the entire BMC. In fact, the 3D BMC structures appear to be the dynamically maintained organelles designed in nature. BMCs present notable structural flexibility and heterogeneity; the mechanical softness of BMC shell structures determined by AFM nanoindentation [30] and the nonequilibrium dynamics of BMC assembly revealed by computational simulations [49] highlighted the differences between BMC and robust virus assemblies. Likewise, the biosynthesis of carboxysomes has been elucidated to correlate with light and chaperons [50, 51]. Very recently, it has been indicated that CcmK3 and CcmK4 can form heterohexamers and cap on the carboxysome shell in a pH-dependent manner, possibly providing a means for regulating carboxysome shell permeability and CO_2_ assimilation in the highly dynamic microenvironment [52]. The exact mechanism underlying how environmental conditions in solution affect the thermodynamic assembly of BMC proteins remains to be investigated, for example, using a combination of experimental studies and computational simulations.

Given the self-assembly of BMC structures, there is a significant interest in engineering BMCs and design of new BMC-based nanobioreactors, molecular scaffolds, and biomaterials in biotechnology applications, for example, enhancing cell metabolism, enzyme encapsulation, molecular delivery, and therapy. Advanced knowledge about the structural resilience and variability of BMCs in response to environmental changes will not only inform strategies for producing robust BMC-based nanostructures in heterologous hosts, i.e., *E. coli* or plants [31, 53, 54], but also pave the way for modulating the formation of 2D nanomaterials as well as the opening and closure of BMC shell-based protein cages, thereby facilitating the functional regulation and targeted molecular delivery. Previously, we have demonstrated the feasibility of using genetic modification approach to manipulate the specific contacts at the interfaces of shell proteins and their self-assembly behaviors [12]. This study strengthens our toolbox for assessing and manipulating BMC shell self-assembly in varying environments.

## Conclusions

In summary, we exploited HS-AFM to carry out the quantitative investigations of BMC shell protein self-assembly under different pH and salt conditions. Formation of larger single-layered patches of shell hexamers was shown to be promoted at 400-mM salt concentration, and neutral pH resulted in a higher dynamic rate of hexamer self-assembly. The organizational transition of shell proteins from hexameric assemblies to fiber-like arrays was also visualized. This study illustrated that environmental conditions play an important role in determining the organization and self-assembly of BMC shell proteins.

## Additional file

Additional file 1: **Table S1.** Mean patch sizes and rates of dynamic events under different environmental conditions. Table S2. Numbers of dynamic events (assembly and disassembly) of Hoch_5815 hexamers in shell sheets under HS-AFM. Figure S1. Schematic representation of sample preparation for AFM. Figure S2. AFM topographs of Hoch_5815 patches formed under different pH ranging from 3 to 10. Figure S3. Difference AFM images of Hoch_5815 captured under varying pH. Figure S4. AFM topographs of Hoch_5815 patches captured in buffers with varied concentrations of CaCl_2_, MgCl_2_, and KCl. Figure S5. Difference AFM images of Hoch_5815 captured under varying salt concentrations. (DOCX 1036 kb)

## Abbreviations

BMC: Bacterial microcompartment
BMC-H: Bacterial microcompartment hexamer
BMC-P: Bacterial microcompartment pentamer
BMC-T: Bacterial microcompartment trimer
DSL: Dynamic light scattering
*E. coli*: *Escherichia coli*
EM: Electron microscopy
HS-AFM: High-speed atomic force microscopy
