# Supplementary material for: Self-Assembly Stability and Variability of Bacterial Microcompartment Shell Proteins in Response to the Environmental Change
Source: Nanoscale Res Lett. 2019 Feb 12;14:54. doi: 10.1186/s11671-019-2884-3 (PMC6372710; doi:10.1186/s11671-019-2884-3)
Supplement: Supplementary file 1 — Table S1. Mean patch sizes and rates of dynamic events under different environmental conditions. Table S2. Numbers of dynamic events (assembly and disassembly) of Hoch_5815 hexamers in shell sheets under HS-AFM. Figure S1. Schematic representation of sample preparation for AFM. Figure S2. AFM topographs of Hoch_5815 patches formed under different pH ranging from 3 to 10. Figure S3. Difference AFM images of Hoch_5815 captured under varying pH. Figure S4. AFM topographs of Hoch_5815 patches captured in buffers with varied concentrations of CaCl2, MgCl2, and KCl. Figure S5. Difference AFM images of Hoch_5815 captured under varying salt concentrations. (DOCX 1036 kb) [file 11671_2019_2884_MOESM1_ESM.docx]

**Additional file 1**

**Table S1 Mean patch sizes and rates of dynamic events under different environmental conditions**. Control refers to images captured under 50 mM Tris-HCl, pH 7.8, 10 mM MgCl_2_. Results are shown as mean ± SD.

| **Conditions** | **Mean patch size**  **(nm^2^)** | **Ratio of test condition to control** | **Rate of hexamer movement**  **(dynamic hexamers nm^-2^ s^-1^)** | **Ratio of test condition to control** |
| --- | --- | --- | --- | --- |
| Control | 705.5 ± 337.6 | 1.00 | 2.0 ± 0.8 | 1.00 |
| pH 3 | 119.8 ± 97.2 | 0.17 | 1.1 ± 0.3 | 0.56 |
| pH 4 | 390.4 ± 282.8 | 0.55 | 1.1 ± 0.3 | 0.56 |
| pH 5 | 529.3 ± 369.1 | 0.75 | 1.5 ± 0.2 | 0.71 |
| pH 6 | 614.4 ± 452.7 | 0.87 | 1.6 ± 0.2 | 0.78 |
| pH 7 | 699.5 ± 255.0 | 0.99 | 2.1 ± 0.3 | 1.04 |
| pH 8 | 784.6 ± 385.7 | 1.11 | 1.9 ± 0.3 | 0.93 |
| pH 9 | 969.7 ± 515.9 | 1.36 | 1.7 ± 0.3 | 0.85 |
| pH 10 | 1221.8 ± 926.5 | 1.73 | 1.6 ± 0.2 | 0.77 |
| 100mM MgCl_2_ | 30.3 ± 5.1 | 0.04 | 2.6 ± 0.7 | 1.27 |
| 200mM MgCl_2_ | 81.3 ± 61.5 | 0.12 | 2.2 ± 0.7 | 1.08 |
| 300mM MgCl_2_ | 722.1 ± 490.3 | 1.02 | 1.9 ± 0.6 | 0.92 |
| 400mM MgCl_2_ | 964.3 ± 695.7 | 1.37 | 1.5 ± 0.6 | 0.77 |
| 500mM MgCl_2_ | 1596.7 ± 1127.8 | 2.26 | 1.3 ± 0.7 | 0.64 |
| 100mM CaCl_2_ | 45.2 ± 13.1 | 0.06 | 4.6 ± 1.3 | 2.24 |
| 200mM CaCl_2_ | 64.0 ± 18.6 | 0.09 | 3.8 ± 1.0 | 1.87 |
| 300mM CaCl_2_ | 2872.0 ± 1731.9 | 4.07 | 1.2 ± 0.5 | 0.61 |
| 400mM CaCl_2_ | 3156.7 ± 2469.6 | 4.48 | 0.8 ± 0.2 | 0.39 |
| 500mM CaCl_2_ | 3673.3 ± 2718.4 | 5.21 | 0.6 ± 0.2 | 0.32 |
| 100mM KCl | 90.3 ± 15.1 | 0.13 | 0.6 ± 0.2 | 0.29 |
| 200mM KCl | 101.3 ± 76.5 | 0.14 | 0.7 ± 0.2 | 0.36 |
| 300mM KCl | 762.1 ± 517.5 | 1.08 | 0.9 ± 0.4 | 0.45 |
| 400mM KCl | 924.3 ± 666.8 | 1.31 | 1.3 ± 0.6 | 0.65 |
| 500mM KCl | 1396.7 ± 851.2 | 1.98 | 1.6 ± 0.5 | 0.77 |

**Table S2 Numbers of dynamic events (assembly and disassembly) of Hoch_5815 hexamers in shell sheets under HS-AFM.** The data are the sums of all the events in the image series for 35 mins (525 frames, 6.4 seconds per frame).

| **Conditions** | **Total dynamic events (*n*)** | **Assembly events (*n*)** | **Disassembly events (*n*)** | **Assembly**  **%** | **Disassembly**  **%** |
| --- | --- | --- | --- | --- | --- |
| Control | 3152 | 1797 | 1355 | 57 | 43 |
| pH 3 | 4372 | 2142 | 2230 | 49 | 51 |
| pH 4 | 4408 | 2336 | 2072 | 53 | 47 |
| pH 5 | 4973 | 2586 | 2387 | 52 | 48 |
| pH 6 | 5033 | 2718 | 2315 | 54 | 46 |
| pH 7 | 5649 | 3220 | 2429 | 57 | 43 |
| pH 8 | 4129 | 2271 | 1858 | 55 | 45 |
| pH 9 | 3541 | 1912 | 1629 | 54 | 46 |
| pH 10 | 3020 | 1600 | 1419 | 53 | 47 |
| 100mM MgCl_2_ | 7314 | 2852 | 4462 | 39 | 61 |
| 200mM MgCl_2_ | 6629 | 3182 | 3447 | 48 | 52 |
| 300mM MgCl_2_ | 6186 | 3464 | 2722 | 56 | 44 |
| 400mM MgCl_2_ | 5542 | 3270 | 2272 | 59 | 41 |
| 500mM MgCl_2_ | 5190 | 3010 | 2180 | 58 | 42 |
| 100mM CaCl_2_ | 8363 | 3763 | 4600 | 45 | 55 |
| 200mM CaCl_2_ | 7971 | 3826 | 4145 | 48 | 52 |
| 300mM CaCl_2_ | 5189 | 2543 | 2646 | 49 | 51 |
| 400mM CaCl_2_ | 4271 | 2520 | 1751 | 59 | 41 |
| 500mM CaCl_2_ | 4016 | 2409 | 1606 | 60 | 40 |
| 100mM KCl | 6739 | 3167 | 3572 | 47 | 53 |
| 200mM KCl | 6215 | 3045 | 3170 | 49 | 51 |
| 300mM KCl | 6084 | 3103 | 2981 | 51 | 49 |
| 400mM KCl | 3176 | 1620 | 1556 | 51 | 49 |
| 500mM KCl | 2944 | 1590 | 1354 | 54 | 46 |


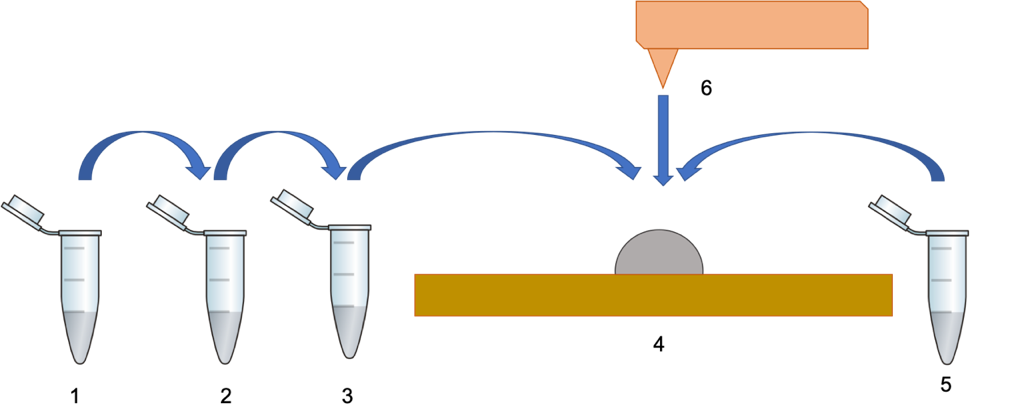
**Fig. S1 Schematic representation of sample preparation for AFM.** Step 1: The stock proteins of HOCH_5815 at approximately 80 mg mL^-1^ in Tris buffer on ice. Step 2: 1 in 40 dilution of HOCH_5815 protein using the experimental buffer on ice. Step 3. 1 in 4 dilution of HOCH_5815 protein using the experimental buffer (final concentration: 0.5 mg mL^-1^) at room temperature. Step 4: 40 μl of samples from step 3 were added on the mica surface and incubate for 5 minutes at room temperature. Step 5: rinse gently the samples on mica three times using the experimental buffer to remove unimmobilized proteins and retain 40 μl buffer on mica surface for imaging. Step 6: AFM imaging of protein dynamics.


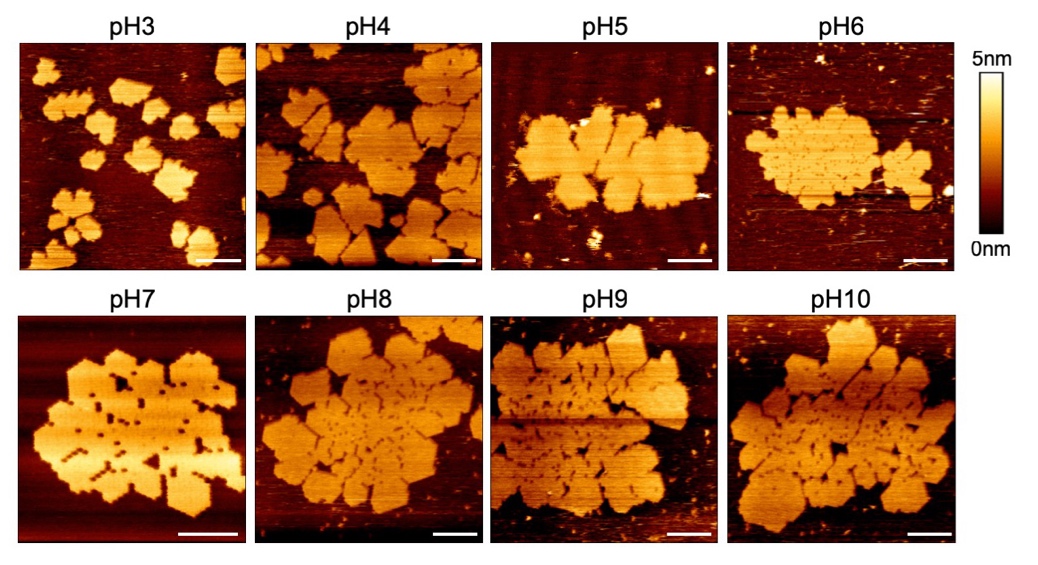


**Fig. S2 AFM topographs of Hoch_5815 patches formed under different pH ranging from 3 to 10.** Analysis of the average size of patches is displayed in Fig. 2a. Scale bar, 50nm.


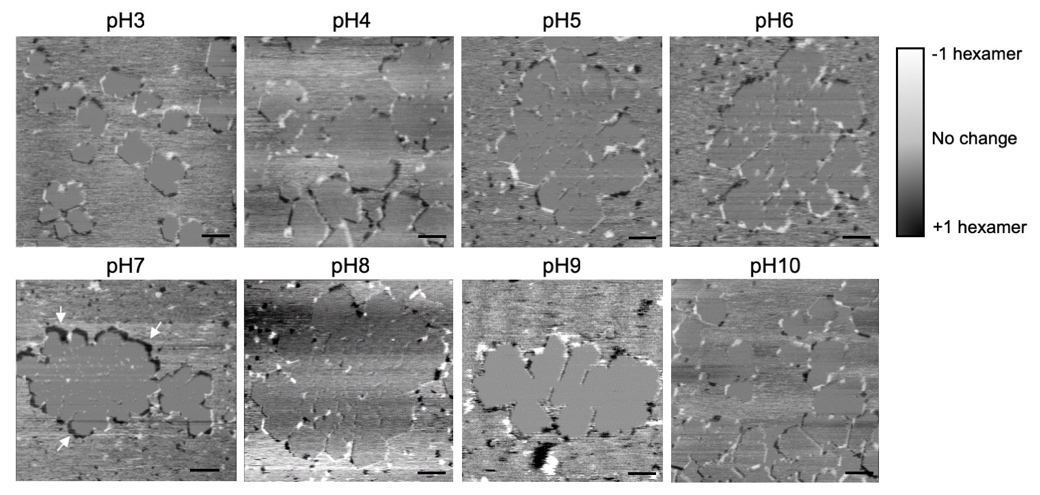


**Fig. S3 Difference AFM images of Hoch_5815 captured under varying pH.** Dark regions represent assembly events of hexamers and white regions represent disassembly events. Analysis of the dynamic rates of Hoch_5815 hexamers in shell patches is shown in Fig. 2b. Scale bar, 50nm.


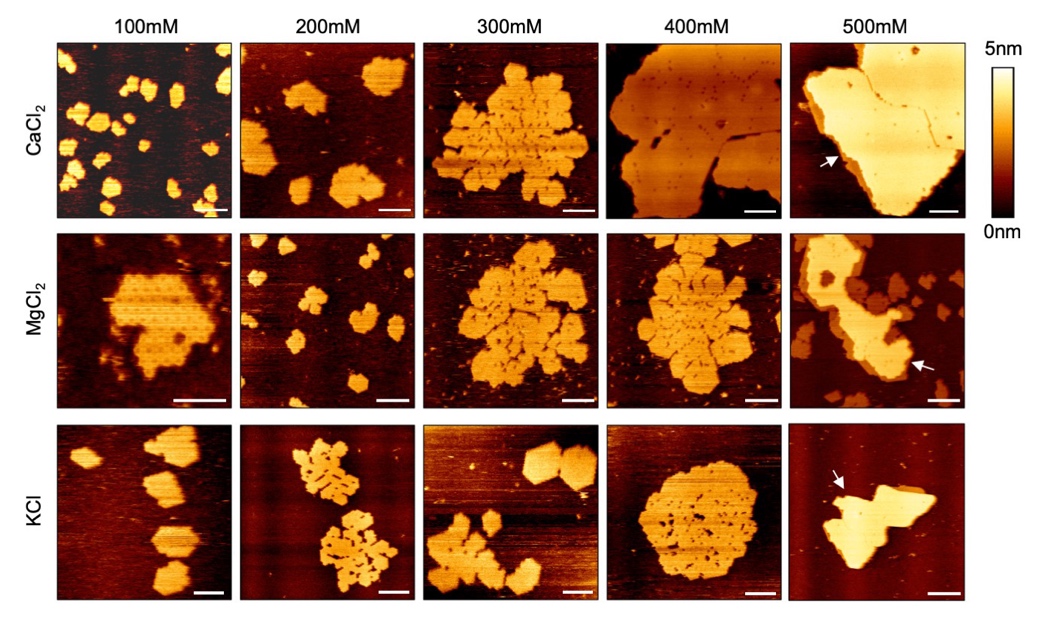


**Fig. S4 AFM topographs of Hoch_5815 patches captured in buffers with varied concentrations of CaCl_2_, MgCl_2_ and KCl.** Analysis of the average size of patches is displayed in Fig. 3a. Scale bars in 100-400 mM represent 50 nm; scale bars in 500 mM represent 500 nm. Arrows indicate double layers of sheets observed under 500 mM salt concentration.


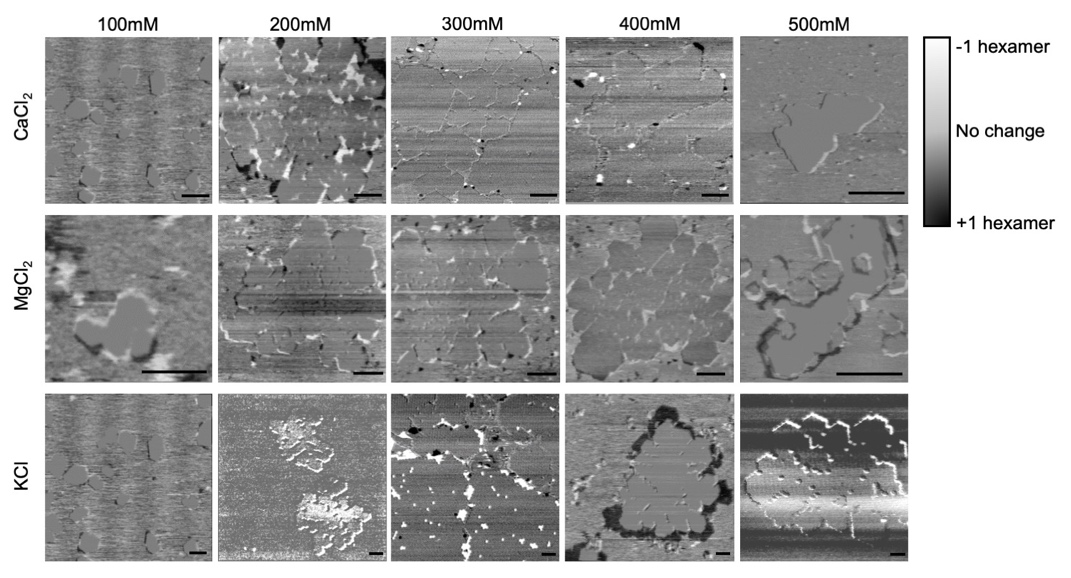


**Fig. S5 Difference AFM images of Hoch_5815 captured under varying salt concentrations.** Dark regions represent assembly events of hexamers and white regions represent disassembly events. Analysis of the dynamic rates of Hoch_5815 hexamers in shell patches is shown in Fig. 3b. Scale bars in 100-400 mM of MgCl_2_ and CaCl_2_ represent 50 nm and scale bars in 500 mM of MgCl_2_ and CaCl_2_ represent 500 nm. Scale bars in AFM images under KCl represent 50 nm.
